# Differential transcriptome regulation by 3,5-T2 and 3′,3,5-T3 in brain and liver uncovers novel roles for thyroid hormones in tilapia

**DOI:** 10.1038/s41598-017-14913-9

**Published:** 2017-11-08

**Authors:** A. Olvera, C. J. Martyniuk, N. Buisine, V. Jiménez-Jacinto, A. Sanchez-Flores, L. M. Sachs, A. Orozco

**Affiliations:** 1 Instituto de Neurobiología, Universidad Nacional Autónoma de México (UNAM), Boulevard Juriquilla 3001, Querétaro, Querétaro, 76230 Mexico; 2 Department of Physiological Sciences and Center for Environmental and Human Toxicology, University of Florida Genetics Institute, Interdisciplinary Program in Biomedical Sciences Neuroscience, College of Veterinary Medicine, University of Florida, Gainesville, FL 32611 USA; 3 UMR 7221, Museum National d’Histoire Naturelle, Centre National de la Recherche Scientifique (CNRS), Sorbone Universités, CP32, Rue Cuvier 57, Paris, 75005 France; 4 Instituto de Biotecnología, Universidad Nacional Autónoma de México (UNAM). Avenida Universidad 2001, Cuernavaca, Morelos 62110 Mexico

## Abstract

Although 3,5,3′-triiodothyronine (T3) is considered to be the primary bioactive thyroid hormone (TH) due to its high affinity for TH nuclear receptors (TRs), new data suggest that 3,5-diiodothyronine (T2) can also regulate transcriptional networks. To determine the functional relevance of these bioactive THs, RNA-seq analysis was conducted in the cerebellum, thalamus-pituitary and liver of tilapia treated with equimolar doses of T2 or T3. We identified a total of 169, 154 and 2863 genes that were TH-responsive (FDR < 0.05) in the tilapia cerebellum, thalamus-pituitary and liver, respectively. Among these, 130, 96 and 349 genes were uniquely regulated by T3, whereas 22, 40 and 929 were exclusively regulated by T2 under our experimental paradigm. The expression profiles in response to TH treatment were tissue-specific, and the diversity of regulated genes also resulted in a variety of different pathways being affected by T2 and T3. T2 regulated gene networks associated with cell signalling and transcriptional pathways, while T3 regulated pathways related to cell signalling, the immune system, and lipid metabolism. Overall, the present work highlights the relevance of T2 as a key bioactive hormone, and reveals some of the different functional strategies that underpin TH pleiotropy.

## Introduction

Thyroid hormones (THs) are endocrine messengers that are well known for their pleiotropic physiological effects in vertebrates. THs regulate development and growth during the early stages of ontogeny, and are required to maintain the energetic balance throughout adulthood^1–3^. Although THs can exert non-genomic effects via membrane bound receptors, they primarily act on the genome by binding with their nuclear receptors (TRs), which function as ligand-dependent transcription factors. This in turn, induces the expression of TH-regulated genes. Compared to other TH metabolites, T3 exhibits the highest affinity for TRs, thus it has been considered the primary bioactive TH^4,5^. However, aside from its well-studied non-genomic effects^6,7^, previous data in teleosts^5,8^ and murine models^9,10^ have shown that 3,5-di-iodothyronine (T2), a product of T3 outer-ring deiodination, is also a transcriptionally bioactive hormone. However, despite this fact, it is greatly understudied when compared to T3. Indeed, we have demonstrated that similar to T3, T2 regulates the transcription of classical TH-regulated genes in the liver of at least two teleost species (killifish: *Fundulus heteroclitus* and tilapia: *Oreochromis niloticus*)^3,8^. The effects of T2 and T3 in the nucleus are mediated by at least two different TRβ1 isoforms that are present in all fish studied to date^5,11^. In the case of tilapia, the effects of T2 are mediated by the long (L-TRβ1) isoform that contains a 9 amino acid insert in its ligand-binding domain, in contrast to the short (S-TRβ1) isoform which lacks this insert and is only activated by T3. In unison, T3 and T2 differentially regulate the hepatic expression of S- and L-TRβ1, respectively *in vivo* and *ex vivo* ^3,5^. These data support the idea that each of these THs exert their biological effects through specific signalling pathways, thus we hypothesize that T3 and T2 can regulate different gene sets, therefore activating specific pathways at the transcriptomic level.

To determine the effect of T2 and T3 on gene regulation, we used an RNA-seq approach and analysed transcriptome modifications after 12 h exposure to T2 and T3 treatment in tilapia cerebellum, thalamus-pituitary and liver. Since T2 nuclear bioactivity has only been studied in the tilapia liver and no data exist for any other tissue, we chose these central nervous system (CNS) regions because THs are well-known modulators of cellular proliferation and neural differentiation in all vertebrates^2^. We show here that T2 plays a significant role, as does T3, in regulating gene transcription. However, T2 specifically regulates gene sets that are involved in pathways that affect particular biological processes, emphasizing its non-redundant role in teleostean physiology.

## Results

### Analysis of Differentially Expressed Genes (DEG)

Samples of the cerebellum, thalamus-pituitary and liver of tilapia that were treated with equimolar doses of T2 or T3 (25 nM) per 12 h (as well as tissues from non-TH treated controls) were sequenced using the Illumina GAIIx platform. We previously showed that this hormone concentration and exposure period does not induce an hyperthyroidal state^8^. Following a quality control check, the resulting short reads were mapped to the tilapia coding sequences (CDS). The statistics for sequencing and mapping are shown in Supplemental Data S1. From the mapping results, we used the effective counts obtained with the eXpress program, to perform differential expression analysis. Gene expression data were obtained through the edgeR bioconductor by comparing the control condition *vs* T2- or T3-treated groups for each tissue (Supplemental Figure S2). A total of 169, 154 and 2863 differentially expressed genes (FDR < 0.05) were detected in the cerebellum, thalamus-pituitary and liver, respectively. All gene expression data from the RNA-seq analysis is provided in Supplemental Data S3.

**Figure 1:**
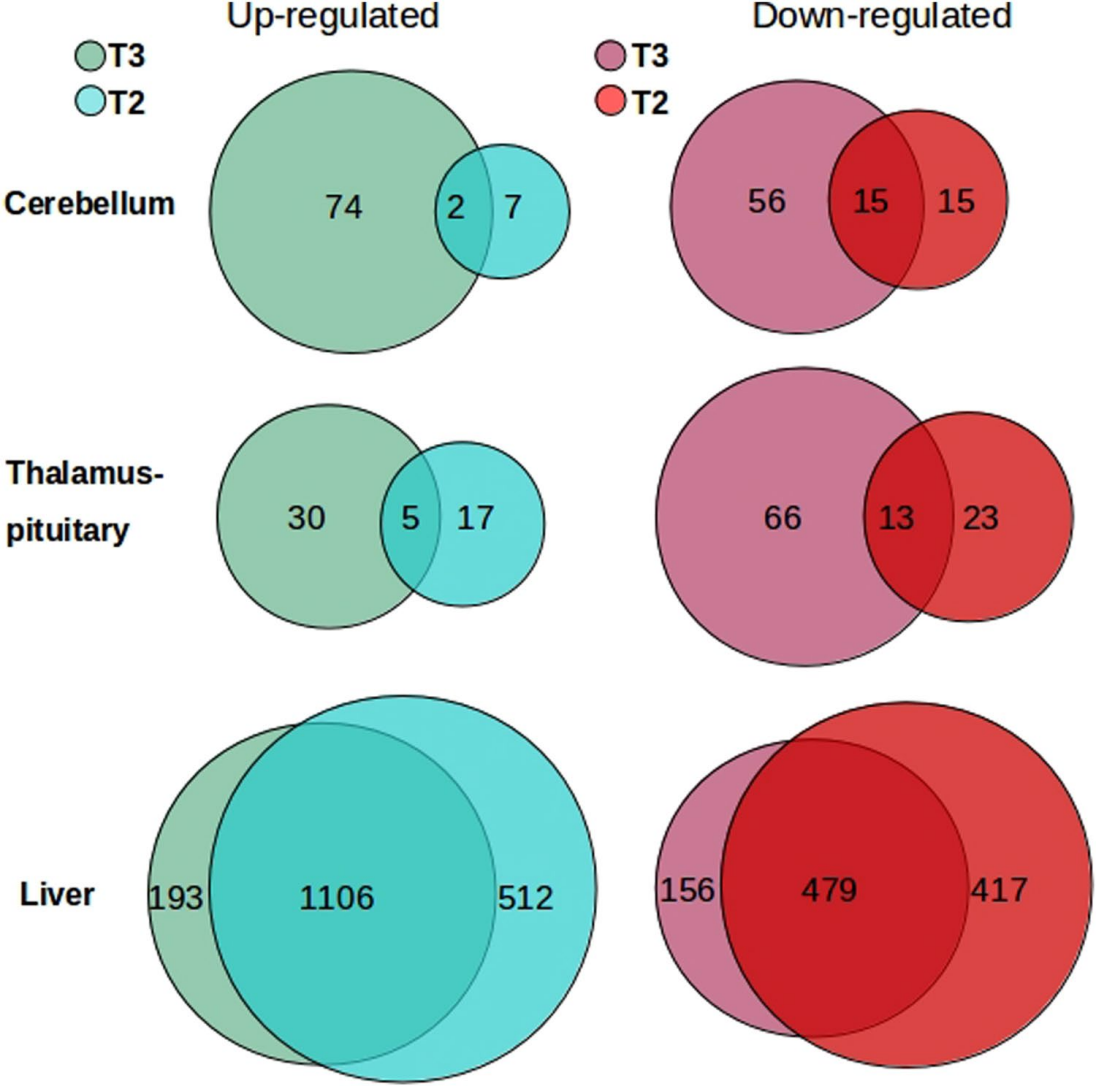
Comparison of TH-regulated genes. Venn diagrams show the number of differentially expressed genes per tissue (FDR < 0.05), up- (green) and down-regulated (red) for T2 and T3. Intersection of circles represents the number of genes regulated by both hormones and the difference represents the genes regulated specifically by either T2 or T3.

We identified a clear difference in the number of differentially expressed genes following T2 and T3 treatments in each tissue. The number of TH-regulated genes were higher in the liver than in the cerebellum or thalamus-pituitary. When comparing T2 and T3 responsive genes, we observed that 130, 96 and 349 genes were uniquely regulated by T3, whereas 22, 40 and 929 were uniquely regulated by T2 in the cerebellum, thalamus-pituitary and liver, respectively. In tissues of the CNS, we observed a greater number of T2- or T3-specific responsive genes compared to those regulated by both hormones (12%), contrary to what was observed in the liver where the majority of genes were regulated by both thyronines (55%). Furthermore, a greater number of genes were regulated by T2 in the liver, while T3 regulated a higher number of genes in the cerebellum and thalamus-pituitary (Fig. 1). In each tissue, we also examined how many T2 or T3 responsive genes were specifically up- or down-regulated. We noted that both thyronines had a tendency to primarily up-regulate genes in the liver, while the hormones down-regulated many genes in the CNS. (Fig. 1).

To confirm and validate some of the results obtained from the differential expression analysis, we validated two genes per tissue that were specifically up-regulated by either T2 or T3 and quantified expression using real-time PCR (RT-qPCR). This independent experiment in tilapia juveniles followed the methods as for RNA-seq (see Materials and Methods). Data were congruent with RNA-seq (Supplemental Figure S4) for luc7-like 1 (LUC7L, p < 0.001) and ubiquitin-specific peptidase 40 (USP40, p < 0.001) following treatment with T2 or T3, respectively, in the cerebellum. Data for anaphase promoting complex subunit 11 (APC11, p < 0.001) and anserinase (ANSN, p < 0.05) were also consistent between RNA-seq and RT-qPCR following treatment with T2 or T3, respectively, in the thalamus-pituitary. The expression level response of sequestosome 1 (SQSTM1, p < 0.001) was also consistent between techniques following treatment with T2 in the liver. However, ATPase H+/K+ exchanging alpha polypeptide (ATPase) mRNA levels did not significantly differ among groups according to qPCR analysis, possibly due to the high biological variability in the samples.

### Expression patterns in response to TH treatments

**Figure 2:**
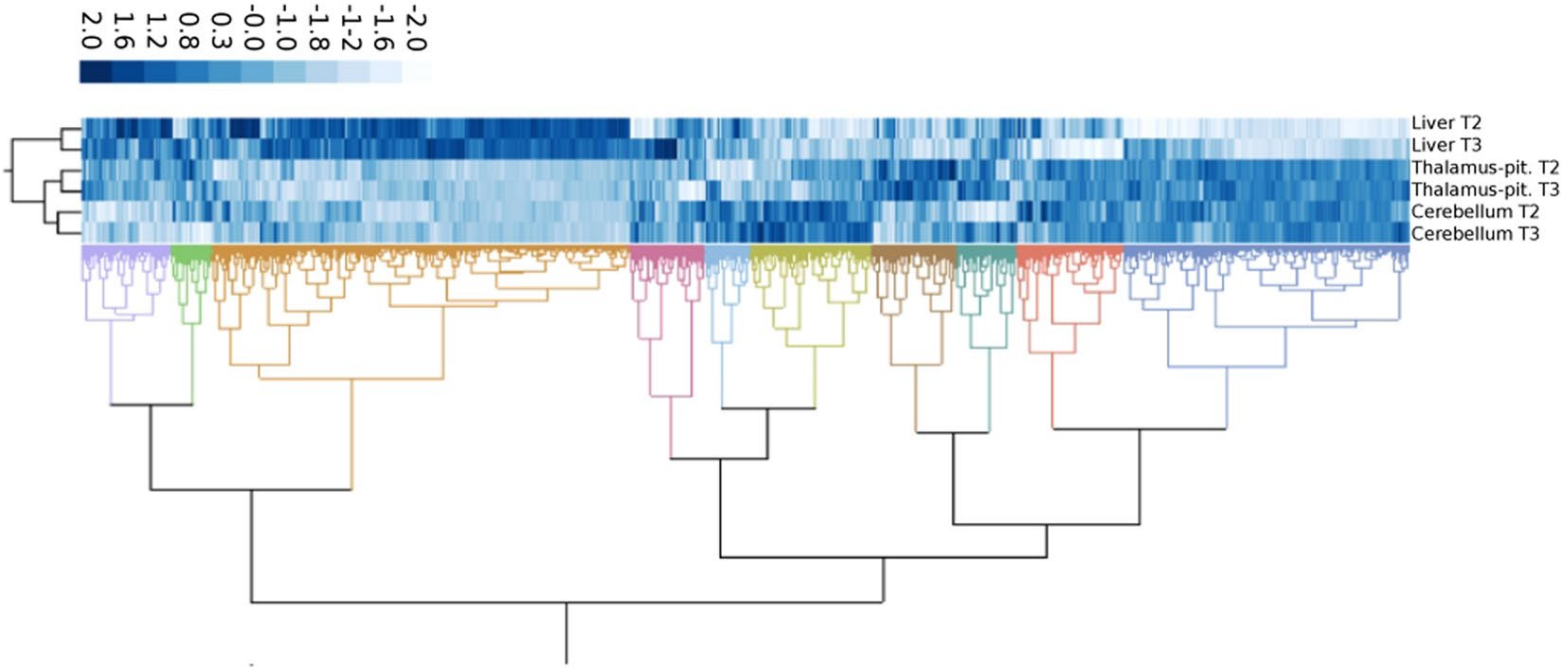
Global expression patterns in response to TH treatments. A heat map of hierarchical clustering conducted with logarithmic fold change of down- (dark blue) and up-regulated genes (light blue) per tissue.

A hierarchical clustering analysis was conducted to determine the global expression patterns of transcripts following T2 and T3 treatment and across tissues (Fig. 2). There were clear patterns of gene expression as a result of the TH-treatments. These patterns were also tissue-specific, and transcripts from the CNS clustered more closely together when compared to the liver which was to be expected.

**Figure 3:**
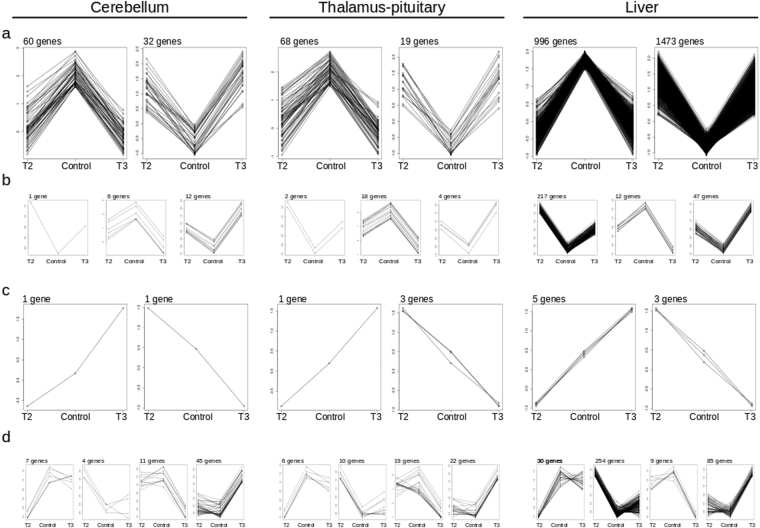
Specific expression patterns in response to TH treatments. Cluster analysis of logarithmic fold change of gene expression normalised and scaled (y-axis), comparing TH treatments versus controls (x-axis). (**a**) Cluster of genes regulated in the same direction, down (left box) or up (right box) by T2 and T3. (**b**) Cluster of genes regulated in the same direction with different magnitude of response. (**c**) Cluster of genes regulated in different directions by T2 and T3. (**d**) Cluster of genes regulated specifically by T2 or T3.

In order to attain a more comprehensive assessment, we performed a cluster analysis using results from DEG. Normalised logarithmic fold change (logFC) of gene expression was used to compare TH treatments versus controls (Fig. 3). The benefit of this approach is to reduce the noise (i.e. reduce influence of false positives and negatives) and to more accurately describe the biological responses. The gene list of clusters can be found in Supplemental Data S5.

As shown in Fig. 3a, we first focused on genes that responded to both treatments. Cluster analysis clearly showed that the total number of TH-regulated genes (up- and down-regulated) was similar in the cerebellum and thalamus-pituitary, while there was a stronger transcriptional response in the liver. Furthermore, the CNS and liver displayed opposite trends, where the majority of these genes were down-regulated in the CNS but up-regulated in the liver. We next focused on the genes that displayed comparable responses, but in which the amplitude of the response was significantly higher to one hormone treatment (Fig. 3b). Four clusters per tissue in which the response of up- and down-regulated genes was higher with either T2 or T3 were identified. These clusters represent a handful of genes in the CNS (~20 genes), and a larger number in the liver (~270 genes). Noteworthy was that the biological response was primarily tissue- and hormone-specific. For example, T3 induced the strongest transcriptional response (down-regulation) in the thalamus-pituitary but not in the liver, and T2 induced the strongest response (up-regulation) in the liver, but not in the other tissues. Cluster analysis also revealed that there were gene clusters that showed opposite regulation after T2 and T3 treatment, although the number of genes in the cluster was limited (Fig. 3c). Finally, a significant number of genes (up to ~380 genes in the liver) displayed specific regulation by one hormone or the other (Fig. 3d), supporting the hypothesis that T2 and T3 regulate specific gene networks in different tissues of teleost fish.

### Transcriptional network analysis

To elucidate thyronine-specific physiological relevance based on expression patterns observed, we identified pathways and gene networks preferentially regulated by T2 and/or T3 by performing gene set enrichment analysis (GSEA) and sub-network enrichment analysis (SNEA). GSEA^12^ and SNEA^13^ are two complementary approaches; GSEA leverages gene ontology and annotated pathways for cell signalling, metabolism, and receptor signalling, while SNEA focuses on gene-gene interactions to build functional networks, resulting in the identification of networks related to a specific biological process. All data from the analysis can be found in Supplemental Data S6 and S7, respectively. For simpler outcome comprehension, results were manually classified into main “themes”. As shown in Tables 1 and 2, we identified pathways in which only T2- or T3-regulated genes were involved (specific pathways). In this context, T2 is mainly involved in pathways related to cell signalling and transcription, while T3 participates in regulating cell signalling, the immune system and lipid metabolism. Pathways regulated by both T2 and T3 include transcription, translation, DNA replication and repair, lipid metabolism, amino acid metabolism, and others (Table 3). Predominantly in the liver, both hormones converge on regulating a given pathway.

**Table 1:** Gene set enrichment analysis of T2-regulated genes. The table shows specific pathways and the main processes for which genes participate in each tissue.

Pathway T2 specific	Main Theme	Cerebellum	Thalamus-p	Liver
Spindle Assembly	Cell growth and death			*
Telomere Maintenance		*		*
Actomyosin-based Movement	Cell motility			*
AdenosineR -> AP-1 signalling	Cell signalling		*	
CholecystokininR -> ELK-SRF signalling		*		
EDG3/5 -> AP-1/ELK-SRF signalling		*		
EndothelinRb -> AP-1/CREB/ELK-SRF signalling		*		
FrizzledR -> CTNNB signalling			*	
GNRHR -> ELK-SRF signalling		*		
ProstaglandinFR -> ATF1/ELK-SRF/CREB signalling		*		
PTAFR -> AP-1/ATF1/CREB/ERK-SRF signalling			*	
Double Strand DNA Non-Homologous Repair	DNA replication and repair			*
NGFR -> NF-kB signalling	Immune system		*	
Transcytosis	Membrane transport		*	
Endosomal Recycling		*		
Capecitabine and Fluorafur metabolism	Other	*		
Irinotecan metabolism		*		
Nicotinate and nicotinamide metabolism				*
Vitamin K metabolism				*
EGFR/ERBB3 -> MEF/MYOD/NFATC/MYOG signalling			*	
CHRAC Chromatin Remodeling	Transcription			*
NURD Chromatin Remodeling				*
NURF Chromatin Remodeling				*
SRCAP Chromatin Remodeling				*
SWI/SNF BRG1/BAF Chromatin Remodeling				*
SWI/SNF BRG1/PBAF Chromatin Remodeling				*

**Table 2:** Gene set enrichment analysis of T3-regulated genes. The table shows specific pathways and the main processes for which genes participate in each tissue.

Pathway T3 specific	Main Theme	Cerebellum	Thalamus-p	Liver
Lysine metabolism	Amino acid metabolism			
Phenylalanine and Tyrosine metabolism		*		
Ubiquitin-dependent Protein Degradation				*
Amino sugars synthesis	Carbohydrate metabolism			*
Galactose metabolism			*	*
Mannose metabolism			*	
L-sugars oxidation			*	
N-Glycan biosynthesis				*
Adherens Junction Regulation	Cell community			*
Gap Junction Regulation		*	*	*
Focal Junction Assembly			*	
Tight Junction Assembly (Occludin)				*
TLR -> AP-1 signalling		*		
TNFR -> CREB/ELK-SRF signaling		*		
Apoptosis	Cell growth and death	*		
Cleavage of Lamina in Apoptosis			*	
AGER -> CREB/SP1 signalling	Cell signalling		*	
AngiopoietinR -> STAT signalling			*	
CannabinoidR -> AP-1/EGR signaling				*
CCR1 -> STAT signalling			*	
CCR2/5 -> STAT signalling			*	
CCR5 -> TP53 signalling			*	
CD19 -> AP-1/ELK-SRF signalling				*
CholinergicRm -> CREB/ELK-SRF signaling				*
EctodysplasinR -> AP-1 signalling		*		
EctodysplasinR -> LEF1 signalling		*		
EGFR -> CTNND signalling			*	
EGFR/ERBB2 -> CTNNB signalling			*	
EGFR -> SMAD1 signalling			*	
EGFR -> ZNF259 signalling			*	
EphrinR -> actin signalling				*
ErythropoietinR -> ELK-SRF/FOS signalling				*
FcIgER -> NFATC1 signalling				*
FGFR1 -> STAT signalling				*
Guanylate Cyclase Pathway				*
IL1R -> STAT3 signalling			*	
IL8R -> CREB/EGR signalling				*
NeuropeptideYR -> ATF/CREB signalling				*
NeurotensinR -> ELK-SRF/AP-1/EGR signalling				*
NTRK -> FOXO/MYCN signalling				*
OxytocinR -> ELK-SRF/GATA/AP-1 signalling				*
SerotoninR1 -> FOS signalling				*
TachykininR -> ELK-SRF signalling				*
ThromboxaneR -> CREB signalling			*	
TNFRSF1A -> STAT signalling				*
VasopressinR2 -> MEF/MYOD/NFATC/MYOG signalling			*	
VEGFR -> NFATC signalling			*	
Single-Strand Nucleotide Excision DNA Repair	DNA replication and repair			*
Pyruvate metabolism	Energy metabolism		*	
Tricarboxylic acid cycle				*
AGER -> NF-kB signalling	Immune system		*	*
Alternative Complement Pathway				*
B-cell receptor -> NF-kB signalling				*
B-cell receptor -> NFATC signalling				*
CD19 -> NF-kB signalling			*	
Classical Complement Pathway			*	
EctodysplasinR -> NF-kB signalling		*		
FibronectinR -> NF-kB signalling			*	
GHR -> NF-kB signalling			*	*
IL12R -> NF-kB/NFATC signalling			*	
IL15R -> NF-kB/NFATC signalling			*	*
IL7R -> FOXO/NF-kB signalling			*	*
Lectin-induced Complement Pathway				*
MacrophageR -> CEBPB/NF-kB signalling				*
Mast Cell Activation				*
NK Cell Activation				*
PTAFR -> NF-kB signalling			*	
T Cell Activation				*
T-cell receptor -> AP-1 signalling				*
T-cell receptor -> NF-kB signalling		*		*
T-cell receptor -> NFATC signalling				*
TLR4/5/7/9 -> NF-kB signalling		*		
Ganglioside-type glycosphingolipid biosynthesis	Lipid metabolism			*
Globoside-type glycosphingolipid biosynthesis				*
Glycosylphosphatidylinositol(GPI)-anchor biosynthesis				*
Metabolism of triacylglycerols			*	
Omega-6-fatty acid metabolism			*	
Sphingolipid metabolism				*
Purine metabolism	Nucleotide metabolism			*
Ascorbate biosyntesis	Other		*	
Extracellular Matrix Turnover			*	
Ethanol metabolism			*	
Gonadotrope Cell Activation				*
Melanogenesis		*	*	*
Skeletal Myogenesis Control		*		*
Histone Ubiquitination	Transcription			*
Lacto- and neolacto-type glycosphingolipid biosynthesis				*
RNA Gene Silencing				*
mRNA Degradation				*
Secretory Pathway: Golgi Transport				*

**Table 3:** Pathways affected by both TH treatments. Enrichment analysis for transcripts regulated by both thyronines. The table shows specific pathways and the main processes for which genes participate in each tissue.

Pathway non-specific	Main Theme	T2 Cerebellum	T3 Cerebellum	T2 Thalamus-p	T3 Thalamus-p	T2 Liver	T3 Liver
Methionine metabolism	Amino acid metabolism			*		*	*
Selencompound biosynthesis						*	*
Tryptophan metabolism						*	*
Cell Cycle Regulation	Cell growth and death					*	*
Centriole Duplication and Separation						*	*
Kinetochore Assembly						*	*
Sister Chromatid Cohesion						*	*
Intermediate Filament Polymerization	Cell motility			*	*	*	
Atlas of signalling	Cell signalling					*	*
EphrinB -> JUN signalling			*	*			
GRM1/5 -> CREB signalling				*			*
Coagulation Cascade	Circulatory system		*		*	*	*
Hedgehog Pathway	Development			*		*	*
Chromosome Condensation	DNA replication and repair		*			*	
Direct DNA Repair						*	*
DNA Replication						*	*
Double Strand DNA Homologous Repair						*	*
Single-Strand Base Excision DNA Repair						*	*
Single-Strand Mismatch DNA Repair					*	*	*
TNFR -> NF-kB signalling	Immune system	*	*				
Biosynthesis of cholesterol	Lipid metabolism					*	*
Metabolism of glycerophospholipids and ether lipids		*			*		
Mevalonate pathway						*	*
Mitochondrial DNA Replication and Transcription	Mitochondrial processes					*	*
Mitochondrial Protein Transport						*	*
Mitochondrion Fusion and Fission					*	*	
Pyrimidine metabolism	Nucleotide metabolism					*	*
Nuclear Envelope	Other				*	*	
Pterine biosynthesis		*	*				
Skeletal Myogenesis Control			*				*
Histone Acetylation	Transcription					*	*
Histone and DNA Methylation						*	*
Histone Phosphorylation						*	*
Histone Sumoylation						*	*
INO80 Chromatin Remodeling						*	*
TRRAP/TIP60 Chromatin Remodeling						*	*
Co-translational ER Protein Import	Translation					*	*
Protein Folding						*	*
Protein Nuclear Import and Export						*	*
Translation						*	*

We also took the approach of determining whether or not there were enriched biological processes found within specific expression profiles (Fig. 3). All results are presented in Supplemental Data S8. While interpreting these data (i.e. clusters), it is important to note that it is based upon the direction of the regulation of participating genes within the cluster. This gene ontology analysis (Table 4) revealed that the cluster of T2/T3 down-regulated genes participates in the majority of enriched pathways within the three tissues, suggesting that there is a redundant response between the hormones that also correlates to the number of differentially expressed genes within this cluster. However, we also found tissue specific responses when we visualized the clusters, for example carbohydrate and energy metabolism were enriched in the liver. Moreover, for gene clusters in which only one thyronine had a regulatory effect, there were also enriched biological processes identified, including cerebellar cell signalling and mitochondrial processes for T3- and T2 down-regulated gene clusters, respectively.

**Table 4:** Pathways affected by clusters of TH-regulated genes. Gene set enrichment analysis for regulated transcripts by clusters. The table shows the main “themes” or processes represented by the transcriptome analysis in each tissue.

	Liver								Thalamus-pituitary								Cerebellum						
	T2/T3 Down	T2/T3 Up	T2 down	T3 down	T2 up	T3 up	T2 down/T3 up	T2 up/T3 down	T2/T3 Down	T2/T3 Up	T2 down	T3 down	T2 up	T3 up	T2 down/T3 up	T2 up/T3 down	T2/T3 Down	T2/T3 Up	T2 down	T3 down	T2 up	T3 up	T2 down/T3 up
Amino acid metabolism	*	*				*			*								*				*	*	
Carbohydrate metabolism	*																						
Cell community	*	*	*		*				*		*						*						
Cell growth and death	*	*	*	*	*		*		*		*	*		*		*	*	*	*	*		*	
Cell motility	*								*				*				*	*					
Cell signalling	*				*								*		*					*			
Circulatory system	*		*						*		*						*						
Development	*								*	*				*			*						
Differentiation	*								*								*						
DNA replication and repair	*	*	*		*				*				*										
Energy metabolism	*	*																					
Immune system	*	*	*	*	*				*								*			*	*		*
Lipid metabolism	*		*				*		*								*	*					
Membrane transport	*	*							*								*						
Mitochondrial processes	*	*																	*				
Nervous system	*								*								*			*			
Transcription	*	*		*	*		*	*	*								*				*		
Translation	*	*			*		*	*	*							*	*		*	*			
Xenobiotics													*										
Other	*	*			*				*	*							*	*		*		*	

## Discussion

Aside from the well-known extra-genomic actions^6,7,14^, data from our group^3,5^ and that of others^10^ support the hypothesis that T2 is a bioactive hormone, capable of acting at the nuclear level by interacting with the TR^3^. This is the first study to elucidate the role of T2 in fish physiology by identifying gene networks whose transcription is affected by T2, either directly or as a product of other transcription factors regulated by TH-treatment.

A strength of the present work is that fish were exposed to equimolar doses of T2 or T3 (25 nM in culture water). Although no hormone concentrations were determined in the present work, in our experience, this hormone concentration, as well as the short (12 h) exposure of the treatment does not induce a hyperthyroidal state^8^. Furthermore, zebrafish intramuscular T3 and T2 concentrations fall within the same range^14^, highlighting the differences between teleosts and mammals in terms of bioactive TH circulating levels. Together, these findings further support the importance of using low equimolar doses in TH treatments. Clear differences in gene regulation were observed, showing that these changes in TH availability were sensed in the liver and the CNS and induced gene expression modulation. TH action is determined by downstream regulators, which include TH transporters, deiodinases, TH receptors, among others. The regulation of these genes is sensitive to changes in TH availability. Unexpectedly, and possibly due to the strict statistical cut-off used, the expression levels of these genes were not significantly different in the RNA-seq data. However, when measured by qPCR in equivalent experimental protocols, genes that determine intracellular TH activation/inactivation (deiodinases 2 and 3, respectively) and transport (MCT8), or TH-dependent gene expression were modulated by T2 and T3 in a tissue-specific manner, further supporting the efficacy of the administration protocol (Supplemental Figure S9).

With the RNA-seq analysis, we identified genes whose expression was regulated by both THs, as well as genes exclusively regulated by T3 or T2. Gene responsiveness to TH treatment was markedly greater in the liver with a total of 2863 regulated genes, when compared with the CNS [cerebellum (169) and thalamus-pituitary (154)] (Fig. 1). This contrast between tissues is not surprising. It reflects in part the difference in TH influx; the liver exhibits a high blood inflow and is a major TH reservoir^15^, while the influx of systemic THs to the CNS is highly regulated in the brain blood barrier (BBB) by TH transporters^15,16^. Another factor that could account for the observed differences is the expression of TRα and TRβ. As depicted in (Supplemental Figure S9), these genes show different expression patterns in tissues of control fish, and are dynamically regulated in a TH- and tissue-specific manner, suggesting an interplay between gene activation or repression. Further studies are required to address this possibility. The differences in gene responsiveness to TH between tissues is consistent with the specific expression of the factors modulating TH signalling.

When visualizing the T2- and T3-expression patterns with cluster analysis, we observed that both hormones were mainly modulating the expression of the same genes and in the same direction (either down- or up-regulation). This redundancy was also observed in HepG2 cells for TRα1 and TRβ1, where these receptors regulated an overlapping set of genes in response to T3^17^. This functional overlap on gene-regulation between T2 and T3 (present study), or in HepG2 cells for TRα1 and TRβ1 could ensure the control of general TH-regulated cell processes, as seen in gene ontology analysis (e.g., lipid metabolism, DNA replication and repair, amino acid metabolism). Interestingly, we observed that some genes showed different amplitudes of response to T2 and T3 (Fig. 3b), while other gene clusters were regulated in opposite directions by these hormones (Fig. 3c). Considering that teleosts express two ligand-specific TR isoforms (T2 + L-TRβ1 or T3 + S-TRβ1)^5,18^, the difference in the magnitude of response for gene transcription could be explained as the recruitment of TR-specific coregulator complexes to the target gene promoters. The fact that other gene clusters are oppositely regulated by T2 and T3 also reflects different molecular mechanisms that favour TH pleiotropism and specificity. This notion can be illustrated by our observations with jun activation domain-binding protein1 (Jab1), which acts as a coactivator in the T2 + L-TRβ1 complex or as a corepressor in the S-TRβ + T3 complex^18^.

Network analysis revealed that a recurring theme that was enriched by both TH treatments, and in each of the three tissues, was that of immune-related processes. In mammals, various groups have described the relationship between the thyroid axis and the immune system, where THs, TSH (thyroid stimulating hormone), and TRH (thyrotropin-releasing hormone) modulate different immune functions in the CNS as well as in peripheral tissues^19–22^. In teleosts, the immunomodulatory role of TH is poorly understood; however, TRα and TRβ have been detected in isolated leukocytes from the head kidney and peripheral blood in the trout (*Oncorhynchus mykiss*), where TRα is higher expressed in immune organs and cells than in liver, whereas TRβ is predominantly expressed in liver, suggesting a tissue-specific function^23^. Furthermore, T3 was found to modulate several immune-related genes and pathways *in vivo*, suggesting complex cross-talk between the thyroid and immune systems that may be conserved among taxa, as well as immune regulation as a novel and previously non-described role for T2 in vertebrates^24^.

We identified transcripts that were specifically regulated by T3 or T2. A significant advance was made in identifying for the first time transcripts that are specifically T2-regulated in the CNS. In the cerebellum for example, among the T2 down-regulated genes are two members of the keratin family KRT1 and KRT2, which are known to be over-expressed in a TH-resistant mouse model^25^. Related pathways involving these proteins included those of cytoskeleton remodelling neurofilaments^26^. Another gene down-regulated by T2, one that is also involved in CNS development, is the poly-ADP-ribosyltransferase Tankyrase, a transcript involved in activating the Wnt signalling pathway. Under normal physiological conditions, this pathway is repressed, leading to appropriate myelination during differentiation of mammalian oligodendrocytes^27^. Lastly, transcription factor SRY-related HMG-box Sox2, is a gene down-regulated by T2, and it is involved in the regulation of embryonic development and in the determination of cell fate. Repression of Sox2 has been described as T3 + TRα1-dependent, promoting neural stem cell commitment and progression toward a migrating neuroblast phenotype in the sub-ventricular zone of adult mice^28^. Together, these results point to T2 as an important CNS modulator, involved in the myelination process, tissue neural differentiation, and in the control of proliferation/differentiation homeostasis. A conserved response was also observed between mammals and teleosts in terms of T3-regulated genes. For example, the gene coding for the synaptosome-associated protein SNAP25 was specifically down-regulated by T3 in both the tilapia cerebellum (present work) and in the developing rat brain, where suppression of SNAP25 transcription is required for proper neurite outgrowth and synaptogenesis^29^. The glial cell adhesion molecule HEPACAM is another gene differentially regulated by T3 in both mammals and teleosts. This gene is involved in myelination, cell motility, and cell-matrix interactions, and HepG2 cells stably expressing TRα and TRβ up-regulate HEPACAM transcription when treated with T3^17^.

Furthermore, in recent work in hyperthyroid mouse liver, 493 genes were identified to be T3-regulated^30^. Notably, among these 493 genes, 124 were identified in our liver transcriptomic data as TH-regulated. Of those, 32 were specifically regulated by T2, 17 by T3, and 75 by both thyronines. In the pool of T2-specific regulated genes, we found cytochrome P450 1 A (cyp1a) and solute carrier family 22 member 7 (SLC22A7) to be differentially expressed in the liver. Both proteins are implicated in the xenobiotic biotransformation system (XBS). Interestingly, these two genes have also been identified as T2-responsive in T2 treated mice^10^. In contrast, of the 493 T3-regulated genes identified in the mouse liver^30^, only 7 were identified as differentially expressed in the tilapia CNS transcriptome, strengthening the hypothesis that tissue-specificity is a wider phenomenon in vertebrates for TH-mediated gene regulation. The multiple observed effects of both T2 and T3 in tilapia were in agreement with a recent transcriptomic analysis in the blue bream, in which T3 treatment elicited pleiotropic effects in liver and brain^31^, supporting the notion that THs act through different functional strategies leading to the modulation of several physiological aspects.

In summary, we demonstrated that, similar to T3, T2 regulates gene expression in the tilapia liver and CNS *in vivo*. Under the conditions of the present work, we identified genes specifically regulated by T2 and T3, and many of these targets constitute novel transcripts that remain uncharacterized in tilapia. The expression profiles observed in response to TH treatment are tissue-specific, and the multiple functions that a gene can elicit are reflected in the many different pathways affected. Furthermore, conserved responses between mammals and teleosts highlight the importance of thyroidal systems for vertebrate homeostasis and support the relevance of T2 action as a key bioactive hormone.

## Materials and Methods

### Biological samples

Tilapia (*Oreochromis niloticus*) that were sexually undifferentiated juveniles ~4–6 g (16 weeks old, corresponding to the developmental stage of 3–4 weeks old pre-puberty mice) were kindly provided by the Quarentine Unit for Tilapia and Catfish at the “Universidad Michoacana de San Nicolás de Hidalgo, México”. Fish were kept for 1 month prior to the experiment in 10 L tanks with aerated freshwater at a temperature of 25 °C on a 12:12 h light:dark cycle and fed twice a day (~40 mg/fish per day) with a commercial diet (Sera Marin, Sera, Germany). All animal experimentation was conducted in accordance with accepted standards of humane animal care, and protocols and procedures regarding handling and euthanasia were reviewed and approved by the Animal Welfare Committee of the Instituto de Neurobiología, UNAM.

### Thyroid hormone treatments

All fish treatments were performed following an immersion protocol routinely used in our laboratory^3^. Treatment conditions were 25 nM of T2 or T3 (SIGMA) previously solubilised in 0.05 M NaOH and 12 h of exposure. This concentration in our experience only elevates intrahepatic T3 levels in less than 10% thus not altering the euthyroidal state^8,32^. To obtain the samples for transcript sequencing, 8 individuals/experimental group were treated by immersion using these experimental conditions. Two biological samples/experimental group were sequenced for liver, cerebellum and thalamus/pituitary. In the case of CNS tissues and due to the small size of the tissues, the biological samples consisted of 2 pools of 4 individuals each. For qPCR analysis, independent groups of fish (12 individuals/experimental group) were treated as described. Three biological samples/experimental group were analysed in duplicate for liver, cerebellum and thalamus/pituitary. For CNS tissues the biological samples consisted of 3 pools of 4 individuals each. In all cases, fish were euthanized, and the tissue samples were quickly removed for total RNA extraction.

### RNA extraction

Total RNA was extracted from brain regions and liver tissues with TRIzol (Invitrogen) according to the manufacturer’s recommendations. RNA purity and integrity was assessed on 1% agarose gels and the concentration was measured with a Nano-Drop ND-1000 UV-Vis spectrophotometer at 260 nm (Nano Drop Technologies). For each treatment (control group and T2- or T3-treated), ~50–70 mg of tissue was used for RNA preparation. Total RNA was extracted and RNase-free Dnase I (Invitrogen) was added to remove residual genomic DNA. RNA integrity was analysed via the Agilent 2100 Bioanalyzer with a minimum RNA integrated numerical value (RIN) of 7 (mean 8.03, SD ± 0.39).

### Preparation of cDNA library and RNA-seq

Libraries were generated using the Illumina TruSeq RNA Sample Preparation Kit according to the manufacturer’s instructions. Transcriptome sequencing was conducted using Genome Analyzer GAIIx (Illumina) at the genome sequencing facility of our university located at “Instituto de Biotecnología-UNAM”. A configuration for pair-end reads with a 72 bp read length was used. GEO accession number GSE96046.

### Bioinformatics analysis

Quality Control (QC) of raw reads was performed using FASTQC software^33^ and contamination and adapter removal was carried out using in-house Perl scripts. QC’ed reads were mapped using the Bowtie 1.1.2^34^ aligner to the annotated reference genome *Oreochromis_niloticus* (Orenil1.0.cds.all, 21,437 coding genes) CDS downloaded using BioMart from Ensembl repository database. Quantification and repetitiveness normalisation were carried out using eXpress software 1.5^35^. Total effective counts for each sample were merged; a matrix was generated using the “abundance_estimates_to_matrix.pl” Perl script included in the Trinity pipeline^36^. DEG analysis was performed using R Bioconductor tool edgeR^37^ through the “run_DE_analysis.pl” script from Trinity and the merged count matrix. Pairwise comparisons among each sample type (Control vs T2 or Control vs T3, each with its respective biological replicate) were performed. To determine DEG, a False Discovery Rate (FDR) of adjusted p < 0.05 was used.

### Cluster analysis

Clustering was carried out with a simple classifier, where normalised expression values are scaled so that the sum of gene expression values across experiments (average between biological replicates) is 0 and standard deviation (SD) equals 1.

1 $$E=\frac{{\rm{x}}-\bar{{\rm{x}}}}{SD}$$

The resulting expression profiles efficiently describe the gene response type independently of their absolute expression values (*i.e*. genes with a similar biological response will be clustered together, despite differences of expression levels). Clusters then correspond to each type of biological response, where genes are up-, down- or not- regulated after T2 or T3 treatment.

### Transcriptome networks and pathway analysis

For pathway analysis, the enrichment p-value cut-off was set at *p* < 0.05 for identifying enriched processes. Pathway Studio V9 (Elsevier) operating with the ResNet 11.0 database was used to obtain further insight into pathways affected by treatment to T2 or T3. This database is built primarily from mammalian data but also contains fish-specific transcripts. Official names for tilapia genes were mapped to the program using homologs for mammals. Although there are clear differences between fish and mammals and there are some genes that will have different/unique/novel roles in species, it is expected that the majority of gene function is conserved in vertebrates and it is much more powerful to leverage all of what we know in vertebrates in terms of gene function and relation, as opposed to a restricted subset (fish-specific data, for which there is vastly less than mammals). Literature evidence to construct relationships between genes and their functions were based largely on mammalian studies but also included evidence from other taxa. Enrichment analysis in Pathway Studio V9 was performed by GSEA and SNEA algorithms using the Mann–Whitney test with an alpha level = 0.05. The analysis used the function “highest magnitude fold change, best p-value”, to filter duplicate probes. These gene sets were defined based on prior biological knowledge of gene ontology and curated pathways. The Curated Reference Pathway Collection included cell process and metabolic pathways and gene ontology categories for biological processes and molecular functions.

### RT-qPCR validation of DEG

The mRNA was reversed transcribed (RT) (M-MLV, Promega) from 2 µg of total RNA using an oligo(dT) primer (final volume of 25 µl). Quantitative PCR was carried out in duplicates in two independent assays using β-Actin as an internal standard^38^ in reactions that contained 1 µl of the RT reaction, 6 µl Maxima SYBR Green/ROX qPCR Master Mix (Fermentas, Waltham, MA, USA), and 500 nM forward and reverse primers in a final volume of 12 µl. PCR protocols and oligonucleotides used for gene amplification are specified in Supplementary Data S10. In all cases, a gene-specific standard curve was used, in a Step One instrument for detection and data analyses according to the manufacturer’s instructions (Applied Biosystems®). For each experimental sample, the mRNA concentration was expressed as molecules per microgram of total mRNA used in the RT reaction (2 µg), obtained by comparison with the standard curve and normalized to the concentration of β-actin. A group of 6 differentially expressed genes (see above) was selected for further validation by RT-qPCR, using criteria that included > 2.5 fold change and an FDR < 0.05, as well as information as to their tissue- and treatment-specificity. All PCR quantifications were carried out in at least two independent assays.

## Electronic supplementary material

Supplemental Data S1, Supplemental Figure S2, Supplemental Figure S4, Supplemental Figure S9 and Supplemental Data S10

Supplemental Data S3

Supplemental Data S5

Supplemental Data S6

Supplemental Data S7

Supplemental Data S8
