# Supplementary material for: Differential transcriptome regulation by 3,5-T2 and 3′,3,5-T3 in brain and liver uncovers novel roles for thyroid hormones in tilapia
Source: Sci Rep. 2017 Nov 8;7:15043. doi: 10.1038/s41598-017-14913-9 (PMC5678081; doi:10.1038/s41598-017-14913-9)
Supplement: Supplementary file 1 — Supplemental Data S1, Supplemental Figure S2, Supplemental Figure S4, Supplemental Figure S9 and Supplemental Data S10 [file 41598_2017_14913_MOESM1_ESM.pdf]

**Supplemental Data S1, Supplemental Figure S2, Supplemental Figure S4, Supplemental Figure S9 and Supplemental Data S10.**

# From: Differential transcriptome regulation by 3,5-T2 and 3',3,5-T3 in brain and liver uncovers novel roles for thyroid hormones in tilapia

Olvera A.[1], Martyniuk C. J.[2], Buisine N.[3], Jiménez-Jacinto V.[4], Sanchez-Flores A.[4], Sachs L.M.[3], Orozco A.[1]

| Supplemental Data S1. RNA-seq statistics | | | | |
|---|---|---|---|---|
| Library | Treatment | Total reads | Mapped reads | Mapped % |
| C1_Cerebellum | | 14514391 | 5755251 | 47.05 |
| C1_Liver | | 4225598 | 1820404 | 49.15 |
| C1_Thalamus-pit | | 14504830 | 5741832 | 47.37 |
| C2_Cerebellum | | 7689739 | 2582045 | 39.92 |
| C2_Liver | | 12107526 | 5612476 | 54.55 |
| C2_Thalamus-pit | Control | 16506206 | 5167001 | 38.43 |
| T2_1_Cerebellum | | 4822691 | 1659861 | 40.56 |
| T2_1_Liver | | 9212482 | 3737020 | 52.34 |
| T2_1_Thalamus-pit | | 13576605 | 4453499 | 40.69 |
| T2_2_Cerebellum | | 13314607 | 5443278 | 48.33 |
| T2_2_Liver | | 10770374 | 4231626 | 49.85 |
| T2_2_Thalamus-pit | T2 | 15314627 | 5075196 | 41.26 |
| T3_1_Cerebellum | | 11232070 | 3701764 | 40.69 |
| T3_1_Liver | | 11823010 | 5713968 | 58.99 |
| T3_1_Thalamus-pit | | 14834362 | 5866975 | 48.29 |
| T3_2_Cerebellum | | 15221025 | 4640154 | 38.14 |
| T3_2_Liver | | 11230279 | 5285284 | 57.91 |
| T3_2_Thalamus-pit | T3 | 10310338 | 3023411 | 37.36 |

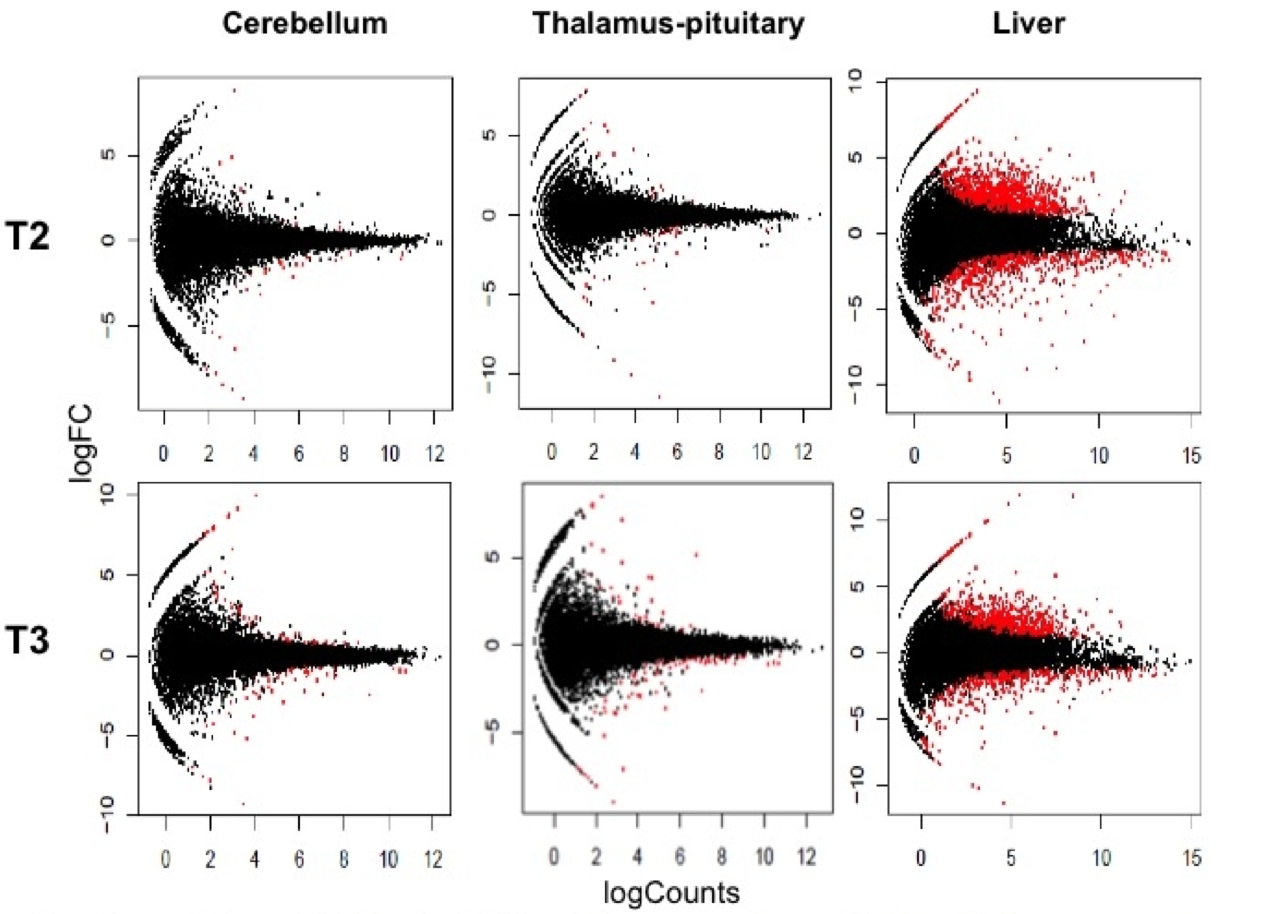


**Supplemental Figure S2. MA plot of differentially regulated genes by T2 or T3.** Data represents individual gene response ploted as logaritmical fold change (*log*FC) vs. total counts (*log*Counts) of mRNA, FDR < 0.05.

**a**

Cerebellum

| T2 LFC | T3 LFC |
|---|---|
| LUC7L 4.39 | - |
| - | USP40 8.02 |

Thalamus-Pit.

| T2 LFC | T3 LFC |
|---|---|
| APC11 3.80 | - |
| - | ANSN 5.12 |

Liver

| T2 LFC | T3 LFC |
|---|---|
| SQSTM1 2.73 | - |
| - | ATPase 11.86 |

**b**

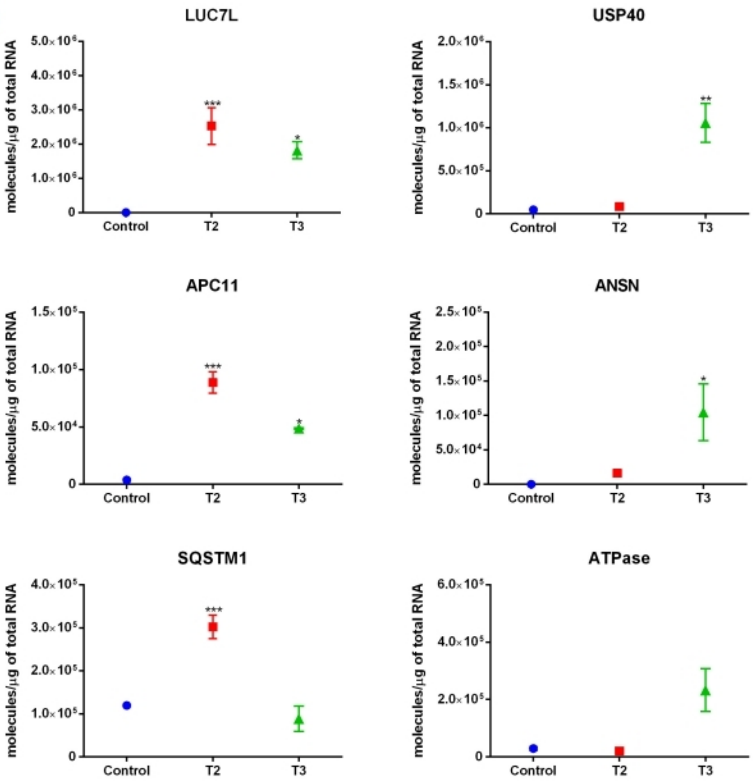


**Supplemental Figure S4. RNA-seq validation by RT-qPCR**. a) Logarithmic fold change (LFC) value of selected genes specifically up-regulated by T2 or T3 in RNA-seq data. b) mRNA expression of selected genes, expressed as molecules per microgram of total RNA. Values are means ± SEM of two independent assays. Significance is indicated *p* = *< 0.01; *p* = **< 0.001; *p* = ***< 0.0001 *vs*. control.

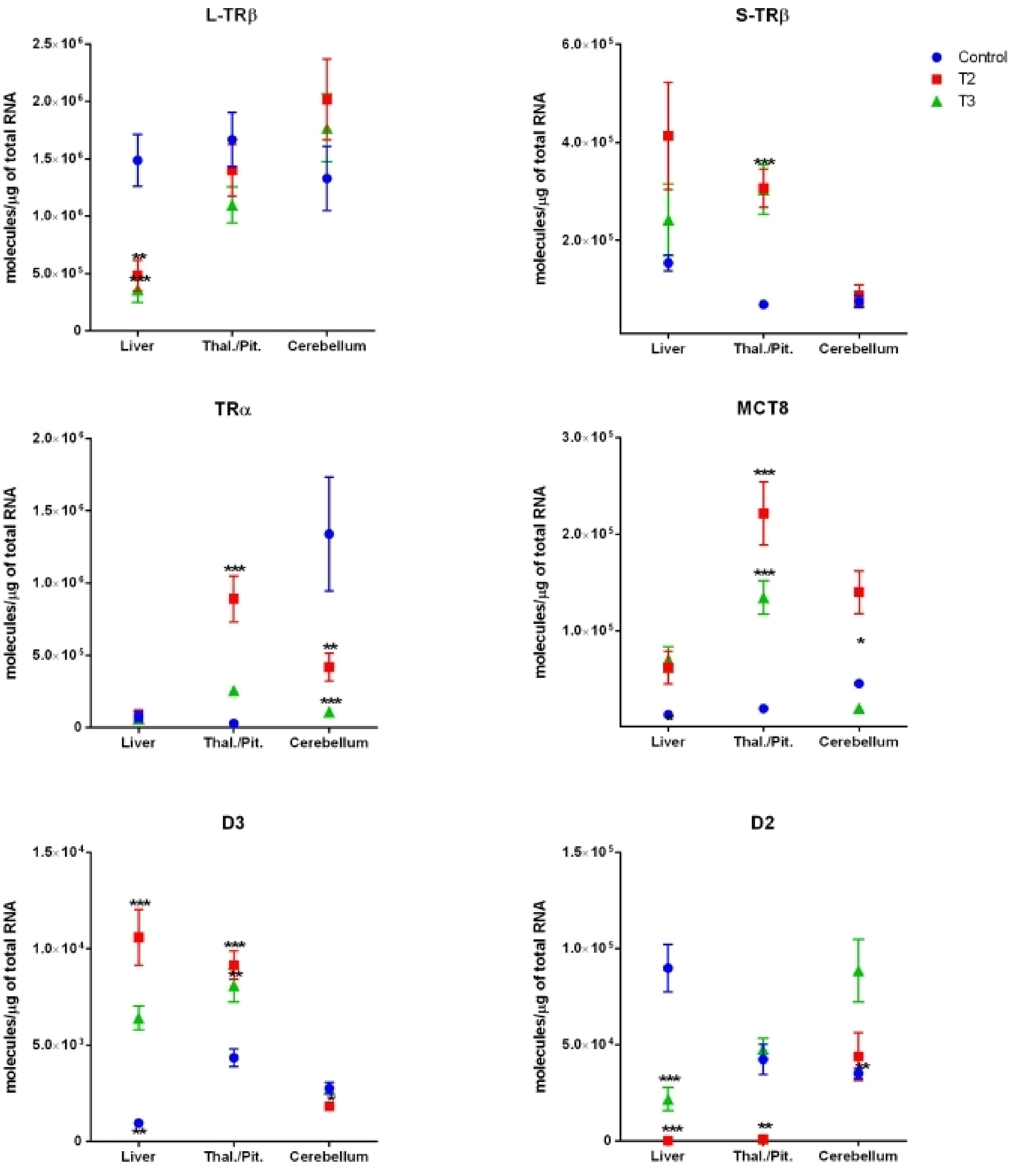


**Supplemental Figure S9. qPCR of genes related to TH signalling**. mRNA expression of selected genes, expressed as molecules per microgram of total RNA. Values are means ± SEM of two independent assays. Significance is indicated *p* = *< 0.01; *p* = **< 0.001; *p* = ***< 0.0001 *vs*. control.

| Supplemental Data S10. PCR protocols and oligonucleotides for qPCR | | | |
|---|---|---|---|
| **gene** | **oligonucleotide 5'--> 3'** | **PCR protocol** | **product** |
| Luc7-like | fw GGAAGCTTCACCTCGGCTT<br>rv TCGTCTCTTTAAGCGCTCCT | 10s at 95°, 10s at 60°C, 10s at 72°C for 40 cycles | 107 bp |
| USP40 | fw CGTCTGCAAGGAGTGTGGAA<br>rv TGCACAGCGGTACAGGTTAT | 10s at 95°, 10s at 60°C, 10s at 72°C for 40 cycles | 154 bp |
| APC11 | fw GGTCAGTGCTCCCACTGTTT<br>rv AGTCATCTCCCGCCTTGAAC | 10s at 95°, 10s at 60°C, 10s at 72°C for 40 cycles | 118 bp |
| Anserinase | fw CCTTCCATCCTGCTGCTCAT<br>rv TTGCTGGAGTCGCTTTCGAT | 10s at 95°, 10s at 60°C, 10s at 72°C for 40 cycles | 134 bp |
| Sequestosome 1 | fw GAGCATACGAGCAGTGAGGG<br>rv CTGTCCCGTCAGGATCAGC | 10s at 95°, 10s at 60°C, 10s at 72°C for 40 cycles | 108 bp |
| ATPase H+/K+ ex. | fw GCCTCCAGTGTCTCATGTGG<br>rv AACAACCGGTTACCACGACA | 10s at 95°, 10s at 60°C, 10s at 72°C for 40 cycles | 141 bp |
| THRB (long isoform) | fw GTGAAGGAAGCTAAGCCTGA<br>rv CACAAGGCAGCTCACAGAAC | 10s at 95°, 10s at 61°C, 10s at 72°C for 40 cycles | 232 bp |
| THRB (short isoform) | fw GCGGAAATTCCTGCCTGAG<br>rv GCAGCTCACAGAACATGGGC | 10s at 95°, 10s at 61°C, 10s at 72°C for 40 cycles | 135 bp |
| THRA | fw GCAGGACTCTAACCCATCA<br>rv GTCATGCTCTTCACCGAACA | 10s at 95°, 10s at 61°C, 10s at 72°C for 40 cycles | 96 bp |
| MCT8 | fw GCTAACGTTCAAGCCTCTGC<br>rv ACTCGGTATGTGACGATGTGA | 10s at 95°, 10s at 61°C, 10s at 72°C for 40 cycles | 134 bp |
| D2 | fw GAAACTTGGCTGTGAGGC<br>rv CTCGTCGATGTAGACCAG | 10s at 95°, 10s at 61°C, 10s at 72°C for 40 cycles | 249 bp |
| D3 | fw GCATCGCTGTTTGGAAGACAG<br>rv TCTCAAAGTAGGCTCCGTACG | 10s at 95°, 10s at 61°C, 10s at 72°C for 40 cycles | 125 bp |
| β-ACTIN | fw ACTTCGAGCAGGAGATGG<br>rv GGTGGTTTCGTGGATTCC | 10s at 95°, 10s at 60°C, 10s at 72°C for 40 cycles | 170 bp |
